# Prevalence, beliefs and impact of drug-drug interactions between antiretroviral therapy and illicit drugs among people living with HIV in Spain

**DOI:** 10.1371/journal.pone.0260334

**Published:** 2021-11-19

**Authors:** Vanessa Castro-Granell, Noé Garin, Ángeles Jaén, Santiago Cenoz, María José Galindo, María José Fuster-RuizdeApodaca

**Affiliations:** 1 Doctoral Programme in Pharmacy, Granada University, Granada, Spain; 2 Department of Pharmacy, Hospital Marina Baixa, Villajoyosa, Alicante, Spain; 3 Department of Pharmacy, Hospital Santa Creu i Sant Pau, Universitat Autònoma de Barcelona, Barcelona, Spain; 4 Instituto de Salud Carlos III, Centro de Investigación Biomédica en Red de salud Mental (CIBERSAM), Madrid, Spain; 5 School of Health Science Blanquerna, Universitat Ramon Llull, Barcelona, Spain; 6 Research Unit, Fundació Docència i Recerca Mutua Terrassa, Terrassa, Universidad de Barcelona, Barcelona, Spain; 7 Medical Department, ViiV Healthcare, Tres Cantos, Madrid, Spain; 8 Spanish Interdisciplinary AIDS Society (Sociedad Española Interdisciplinaria del Sida, SEISIDA), Madrid, Spain; 9 Department of Infectious Diseases, Hospital Clínico Universitario, Valencia, Spain; 10 Department of Social and Organizational Psychology, Universidad Nacional de Educación a Distancia (UNED), Madrid, Spain; University "Magna Graecia" of Catanzaro, ITALY

## Abstract

Drug use implies important challenges related to HIV management, particularly due to an increased risk of potential interactions between antiretroviral therapy (ART) and illicit drugs (pDDIs). This study analyses the prevalence and severity of pDDIs among people living with HIV (PLHIV). It also explores their awareness of pDDIs and their beliefs about the toxicity that they may cause, as well as the impact of pDDIs on selected health variables. We conducted an on-line cross-sectional survey across 33 Spanish hospitals and NGOs to collect demographics and clinical data. pDDIs were checked against the Interaction Checker developed by Liverpool University. The sample of the present study was composed of 694 PLHIV who used illicit drugs. They represented 49.5% of the 1,401 PLHIV that participated in the survey. After excluding 38 participants due to lack of information on their ART or illicit drug use, 335 (51.1%) participants consuming drugs presented with some potentially significant pDDIs between their ART and illicit drugs, with a mean of 2.1±1.7 (1–10) pDDIs per patient. The drugs most frequently involved in pDDIs were cocaine, cannabis, MDMA and nitrates ("*poppers*"). The prevalence of pDDIs across ART regimens was: protease inhibitors (41.7%); integrase inhibitor-boosted regimens (32.1%), and non-nucleoside reverse transcriptase inhibitors (26.3%). An awareness of pDDIs and beliefs about their potential toxicity correlated positively with intentional non-adherence (p<0.0001). Participants with pDDIs exhibited a higher prevalence of intentional non-adherence (2.19±1.04 vs. 1.93±0.94; *p* = 0.001). The presence of pDDIs was not associated with poorer results in the clinical variables analysed. A significant proportion of PLHIV who use drugs experience pDDIs, thereby requiring close monitoring. pDDIs should be considered in the clinical management of HIV patients. Adequate information about pDDIs and indicators about how to manage ART when PLHIV use drugs could improve ART non-adherence.

## Introduction

There are currently 38 million people living with HIV (PLHIV) worldwide, of whom 67% have access to antiretroviral therapy (ART) [1]. In Spain, the prevalence of PLHIV is estimated at around 0.3% in the general population. Over 90% of them are on ART [2]. In most developed countries, where these treatments are widely available, combined ART has resulted in increased life expectancy among PLHIV [3–6] and in the chronification of the infection. Despite the success of highly active ART, new challenges have arisen that could lead to treatment failure. Prominent among such challenges is the appearance of interactions between ART drugs and medications or illicit drugs taken concomitantly with them.

ART agents pose a high risk for potential drug-drug interactions (pDDIs) mainly induce, inhibit, or are a substrate of P450 cytochrome enzymes, particularly isozyme CYP3A4 [7–9]. These common metabolic pathways may lead to pDDIs in PLHIV who use illicit drugs and are on ART. However, while the prevalence of pDDIs associated to medication used concomitantly with ART because of associated comorbidities has been well described, with reported rates ranging between 34.9% and 89.2% [3, 10–13] there is still a dearth of data on pDDIs between ART drugs and illicit drugs [14–17].

It should also be considered that not all ART or illicit drugs are associated with a high risk of pDDIs. Certain ARTs, such as boosted-protease inhibitor-based regimens (bPIs), some non-nucleoside/nucleotide reverse transcriptase inhibitors (NNRTIs), and the co-formulation including elvitegravir/cobicistat, which belong to the boosted integrase strand transfer inhibitor (INSTI) therapeutic group, may induce or inhibit the metabolism of drugs taken concomitantly [14]. Conversely, nucleoside/nucleotide reverse transcriptase inhibitors (NRTIs), NNRTIs such as etravirine, doravirine and rilpivirine, non-boosted INSTI like raltegravir, dolutegravir, bictegravir and cabotegravir, and the fusion inhibitor maraviroc are characterised by a low pDDIs potential and are considered safer for concomitant use with illicit drugs [8, 14, 18]. Moreover, illicit drugs are also characterised by a variable risk of pDDIs. Cannabis, opioids, and nitrates seem to present with a low pDDIs potential with ART drugs [19], while GHB/GBL, ketamine, MDMA, methamphetamines and mephedrone are associated with a higher potential.

According to the results of our previous research [20], the prevalence of illicit drug use among PLHIV is considerable in Spain, exceeding 50% in men who have sex with men (MSM). PLHIV who use illicit drugs may be classified into four clear epidemiological patterns of consumption [20]. The first two, comprised mostly of heterosexuals (HTX), tend to consume traditional drugs such as heroin, exhibit poorer adherence to ART, and display worse health outcomes. The other two, made up mostly of MSM, tend to consume recreational drugs, show a high rate of polydrug use, and present with an increased risk of sexually transmitted infections (STIs). These patterns of consumption shown by PLHIV who use illicit drugs may be associated with various pDDIs risk patterns. This has scarcely been explored in the literature, yet it could be instrumental in helping to predict which consumption patterns may be related with a higher prevalence of pDDIs, thereby allowing greater anticipation and more effective ART planning to prevent therapeutic failure.

pDDIs may have important clinical consequences in the context of patients on ART who use illicit drugs. Firstly, it may boost the toxicity of illicit drugs and/or decrease the efficacy of the medications involved, which could result in treatment failure and in the exhaustion of therapeutic options [16, 19, 21]. Also, induction of the metabolism of illicit drugs may inhibit their "desired" effect, thus setting off a cycle whereby users are driven to combine a greater number of substances, increase the dose of the drugs consumed, or even resort to the parenteral route to bypass the first pass mechanism associated with the oral route, achieving the desired rush at the expense of higher and more unpredictable toxicity risks [14, 16]. Some published studies have reported serious or even lethal pDDIs between illicit drugs and ART [14, 16, 17, 22–24].

Furthermore, pDDIs have been found to be related to a decreased adherence to ART. PLHIV who use drugs may deliberately stop taking their medication when consuming alcohol or drugs, believing that this could protect them from interaction-derived toxicity. These people often find themselves in the dilemma of either continuing with their ART or giving up drug consumption [25]. Inadequate adherence could impair the benefits of treatment [26, 27]. The few studies looking into intentional non-adherence show that patients who deliberately fail to take their medication exhibit poorer adherence-related results [25] than those who forget to take their medication or those who experience drug intoxication (non-deliberate lack of adherence). The belief that pDDIs results in toxicity seems to be widespread among PLHIV who use illicit drugs [25].

A potential negative impact on health economics could also be expected, as it has been noted that illicit drug consumption is associated with a higher incidence of visits to the emergency room and with more hospital admissions [28, 29]. Nevertheless, no studies have so far determined whether the presence of pDDIs could play a role on that front.

Thus, there is a need to gain a better understanding of the pDDIs that PLHIV who use illicit drugs may be exposed to as a result of their consumption patterns and their status regarding a series of health variables. With this aim, our study set about performing a secondary analysis of PLHIV who use illicit drugs in the study of Fuster-RuizdeApodaca et al. [20] in order to: (1) assess the prevalence and severity of pDDIs in PLHIV who use illicit drugs receiving ART; (2) analyse these pDDIs across the consumption patterns; (3) explore the awareness and beliefs of PLHIV regarding the potential for pDDIs and the toxicity they may cause, and how they influence adherence; and (4) evaluate the implications of pDDIs for health and healthcare resource-related variables.

## Material and methods

### Design and sample

**Fig 1:**
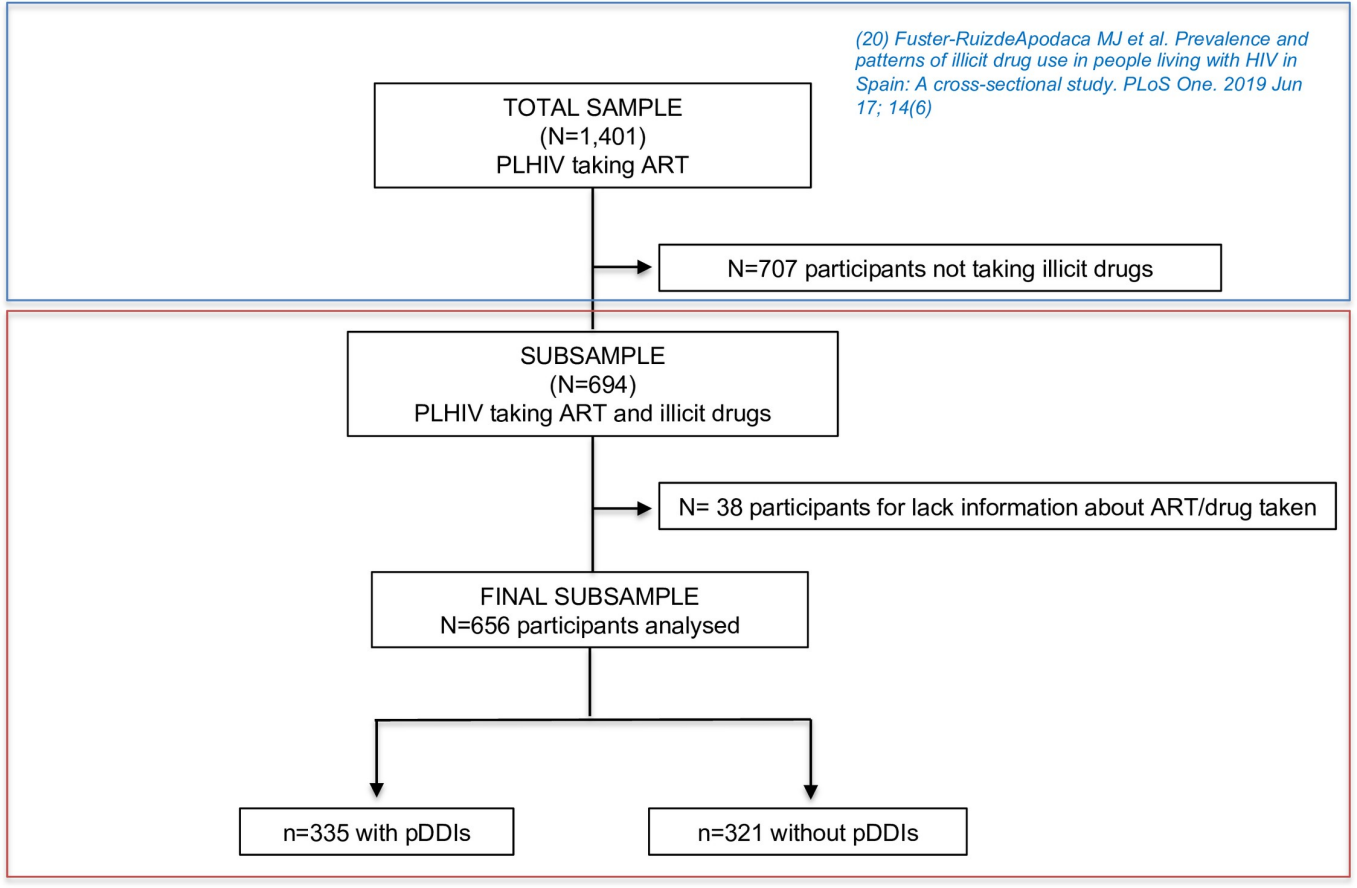
Study sampling. PLHIV: people living with HIV; ART: antiretroviral therapy; pDDIs: potential drug-drug interactions; N: patients; n: number of pDDIs.

This study is part of a broader research project aimed at analysing several aspects of illicit drug use by PLHIV in Spain. The sub-sample used in the present study came from an observational cross-sectional ex-post-facto study that surveyed 1,401 PLHIV [20] recruited by convenience sampling (Fig 1). Data were collected through an online survey between November 2016 and May 2017. Inclusion criteria were: age ≥18 years, HIV infection, ART for at least one year, and an absence of any severe psychiatric or cognitive disorders. The present paper performed a secondary analysis using sub-sample of PLHIV who used drugs (n = 694). A previous qualitative phase of the study included interviews with 21 PLHIV who use illicit drugs to develop the questionnaire [28]. A community-based participatory research paradigm was used, involving individuals from the population under study at all research phases [30, 31]. A group of experts from several social science and healthcare fields guided the research and participated in its different stages.

### Procedures

Data were collected across 12 Spanish regions (12 hospitals and 21 NGOs) from 36 different institutions. The Interdisciplinary Spanish AIDS Society (SEISIDA) performed and coordinated the study. A series of collaborating healthcare providers were in charge of recruiting the participants during their clinical visits. They were given instructions on the procedures of the online self-administered survey through a tablet device. Completion of the survey took about 40 minutes. The survey response rate was 93.4% (82–100%). The main reasons argued for non-completion were not having enough time, the length of the survey, visual impairment, or lack of tablet skills.

### Ethics statement

Participants were informed of the study and asked to provide written informed consent. The Ethics Committee of the Valencia Clinical Hospital approved the research protocol. All investigators worked according to the principles expressed in the Declaration of Helsinki.

### Measures

Procedures regarding the survey design are described in Fuster-RuizdeApodaca et al. [20]. The variables used in the present analysis included:

#### Use of illicit drugs and other substances

The following variables were used: type, frequency and route of consumption during the last year. A list of 18 illicit drugs was included in the survey.

#### pDDIs between illicit and ART drugs

Theoretical pDDIs between illicit drugs and ART regimens were checked against the HIV Drug Interaction Checker, developed by the University of Liverpool (http://www.hiv-druginteractions.org) [18]. The analysis considered only those pDDIs that may require close monitoring or alteration of drug dosage or timing of administration, and contraindicated combinations (due to their potential to cause serious adverse events) [7].

#### Awareness, beliefs, intentional non-adherence, and communication with healthcare providers on the use of drugs and potential pDDIs

The following variables were measured: (i) patients awareness of the potential pDDIs (two items), (ii) beliefs about the potential toxicity derived from those pDDIs (two items), (iii) intentional non-adherence to ART when using illicit drugs (four items), and (iv) communication with healthcare providers on the use of illicit drugs and pDDIs (four items). As the items under each construct showed adequate reliability, their composite mean values were used in the analyses. All items were rated on a 5-point scale (1: ’*not at all*’, 5: ’*a lot*’). Items were designed based both on the results of the previous qualitative study conducted in phase 1 of this research [28] and on the literature review. Many of them were adapted from those used by Kalichman [25, 32].

#### Adherence to ART

We used version 2.0 of the Questionnaire to Evaluate Adherence to HIV Therapy developed by Remor et al. [33]. The questionnaire comprises 17 items rated on a 5-point scale. The sum score of all the items was calculated, with higher scores indicating higher adherence to treatment. Reliability of the questionnaire was adequate (Cronbach’s α = 0.78).

#### Health-related quality of life

The Spanish validated version of the WHOQOL-HIV-BREF questionnaire was used [34]. The survey contains 31 items distributed over six domains. A general domain was used to measure overall perception of health-related quality of life [35]. Items are rated on a 5-point scale. Negative items were reversed-coded. Higher scores across the different items indicate better quality of life.

#### Health and use-of-healthcare-system-related variables

The following self-reported questions related to the participants’ health status were included: time from HIV diagnosis, time on treatment and virological and immunological data (most recent viral load and CD4 measurements). The survey also contained three items asking about the number of visits paid to different health services (outpatient clinic, emergency room, hospital wards) during the last year. Possible responses ranged from 1 ’*much less frequently than usual*’ to 5 ’*much more frequently than usual*’.

The survey also collected several socio-demographic data: age, gender, sexual orientation, educational level, employment status, financial resources, and city of residence.

## Data analysis

Descriptive analysis of the sample included frequencies, proportions, means, ranges and SDs, as appropriate. Firstly, we quantified the prevalence of potential relevant pDDIs across the type of illicit drug used and type of ART regimen. Subsequently, we described and compared pDDIs according to the drug use pattern and the type of illicit drug or ART regimen used. The illicit drug use patterns in the sample were analysed in a previous study (Fuster-RuizdeApodaca et al. [20]).

Secondly, we described the participants awareness, interactive beliefs, non-intentional adherence, and communication with healthcare providers about the use of illicit drugs. Pearson’s correlations were used to study the associations between these variables. Then, differences in intentional non-adherence were compared between participants with and without pDDIs, controlling for the effect of their awareness of potential pDDIs and their beliefs about the toxicity resulting from them through an analysis of covariance (ANCOVA).

Finally, either Student’s *t* test or the chi-squared test was used to analyse the differences across other health-related variables, depending on the nature of the data. The results were checked with nonparametric Mann-Whitney U test. Statistical significance was set at *p* value <0.05.

The analyses were performed using the SPSS v.22 software.

## Results

### Participants’ characteristics

Participants were mainly MSM, with a mean age slightly above 44 years. They were all Spanish nationals living in urban areas (Table 1).

**Table 1:** Participants’ socio-demographic and HIV-related characteristics.

	% (N)^1^
Socio-demographic characteristics	100 (694)
Age, mean (*SD*) (years)	44.59 (9.76)
Gender	
Male, % (n)	86.2 (598)
Female, % (n)	11.8 (82)
Transgender, % (n)	2.0 (14)
Sexual orientation	
Heterosexual, % (n)	34.3 (238)
Homosexual, % (n)	62.7 (435)
Other, % (n)	3.0 (21)
Transmission route	
Sexual contact % (n)	65.9 (457)
Intravenous drug use % (n)	23.3 (162)
Other % (n)	10.8 (75)
Current relationship	
Yes, % (n)	29.4 (204)
No, % (n)	70.6 (490)
Educational level	
No studies, % (n)	4.3 (30)
Primary, % (n)	26.5 (184)
Secondary, % (n)	32.4 (225)
University degree, % (n)	33.4 (232)
Other, % (n)	3.3 (23)
Work situation	
Working, % (n)	51.2 (355)
Unemployed, % (n)	20.9 (145)
Retired or disability, % (n)	19.6 (136)
Other, % (n)	8.4 (58)
Monthly incomes	
None	13.3 (92)
≤ 1,000 € % (n)	36.0 (250)
1,000–1,500 € % (n)	30.7 (213)
1,500–2,000 € % (n)	7.9 (55)
> 2,000 € % (n)	12.1 (84)
Country of birth	
Spain % (n)	81.7 (567)
Europe % (n)	4.9 (34)
Outside of Europe % (n)	13.4 (93)
Residence^a^	
Rural, % (n)	4.5 (31)
Urban, % (n)	95.1 (660)
Unknown % (n)	0.4 (3)
HIV related variables	
Time diagnosed, *M±SD* (years)	14.54 (9.82)
Time on ART, *M±SD* (years)	11.84 (8.34)
CD4 cell count	
< 200 CD4 cells/μL % (n)	4.3 (30)
200–400 CD4cells/μL % (n)	8.2 (57)
> 400 CD4 cells/μL % (n)	68.9 (478)
Unknown % (n)	18.6 (129)
Viral load	
Undetectable^b^ % (n)	90.6 (629)
Detectable % (n)	5.5 (38)
Unknown % (n)	3.9 (27)

Note

^1^Data provided in frequencies, and percentages except when other statistics are indicated.

^a^ "Urban" when more than 10,000 inhabitants.

^b^Undetectable viral load was defined as <50 copies/ml. ART: antiretroviral therapy.

### Prevalence of pDDIs in PLHIV who used illicit drugs

After excluding 38 participants due to a lack of information on their ART or illicit drugs, 335 (51.1%) of participants consuming drugs exhibited pDDIs between their ART regimen and the drugs they were consuming or had consumed in the previous year. Specifically, a total of 708 significant or contraindicated pDDIs were identified. Only one of the pDDIs detected was considered a contraindicated combination. The mean number of pDDIs per patient was 2.1±1.7 (range 1–10).

A total of 33.5% (n = 220) of participants consumed a single illicit drug, 19.5% (n = 128) consumed two, 14.2% (n = 93) consumed three, 7.3% (n = 48) consumed four, and the remaining 25.5% (n = 167) consumed five or more drugs.

**Fig 2:**
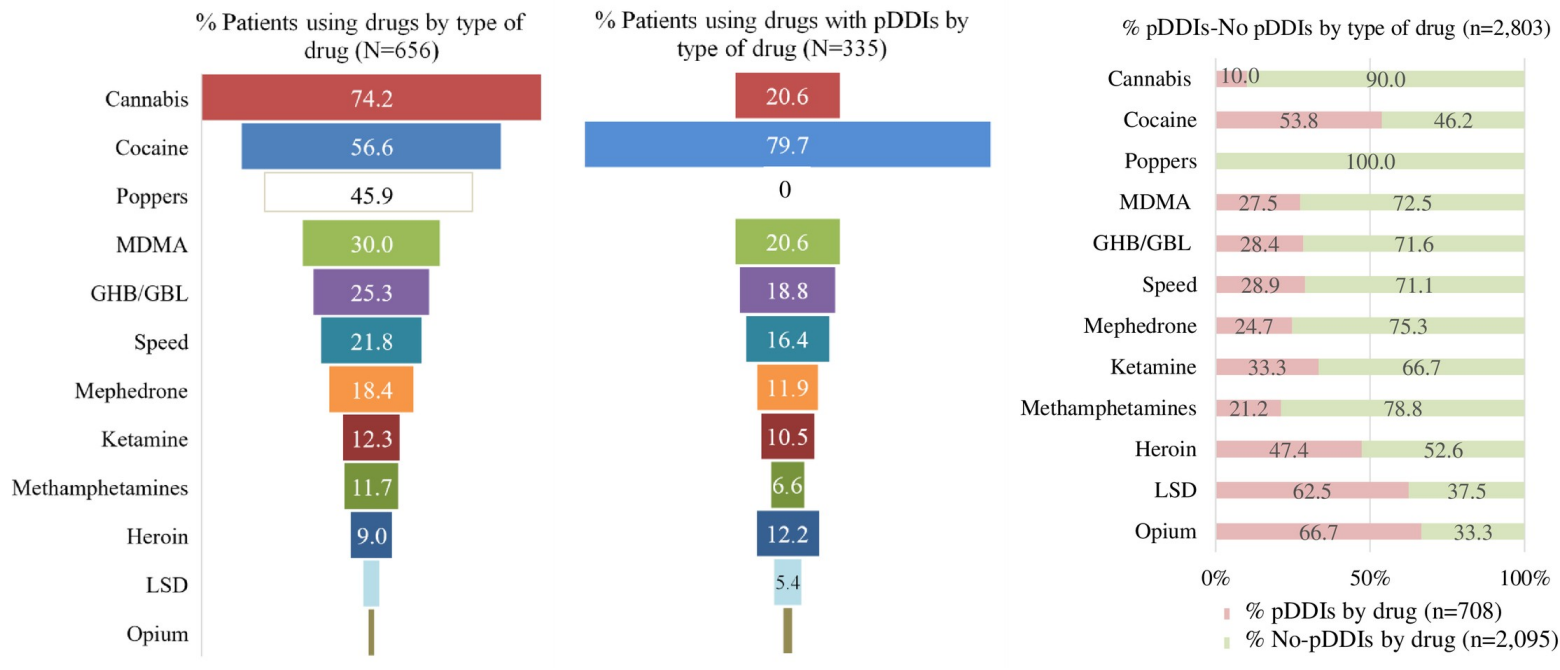
Prevalence of participants using drugs, with potential interactions and distribution per type of drug. MDMA: 3,4-Methylenedioxymethamfetamine; GHB/GBL: gamma-hydroxybutyrate/gamma-butyrolactone; LSD: Lysergic acid diethylamide; pDDIs: Potential drug-drug interactions; No-pDDIs: No potential drug-drug interactions; N: patients; n: number of pDDIs.

The most commonly used illicit drugs included cannabis, cocaine, nitrates ("*poppers*") and MDMA (Fig 2). The ones exhibiting a higher prevalence of pDDIs were cocaine, cannabis, MDMA, and GHB/GBL. The only drug that did not result in pDDIs was nitrates ("*poppers*"). The higher pDDIs:non-pDDIs ratios corresponded to opioids (2:1), LSD (1.7:1), cocaine (1.2:1) and heroin (0.9:1).

**Fig 3:**
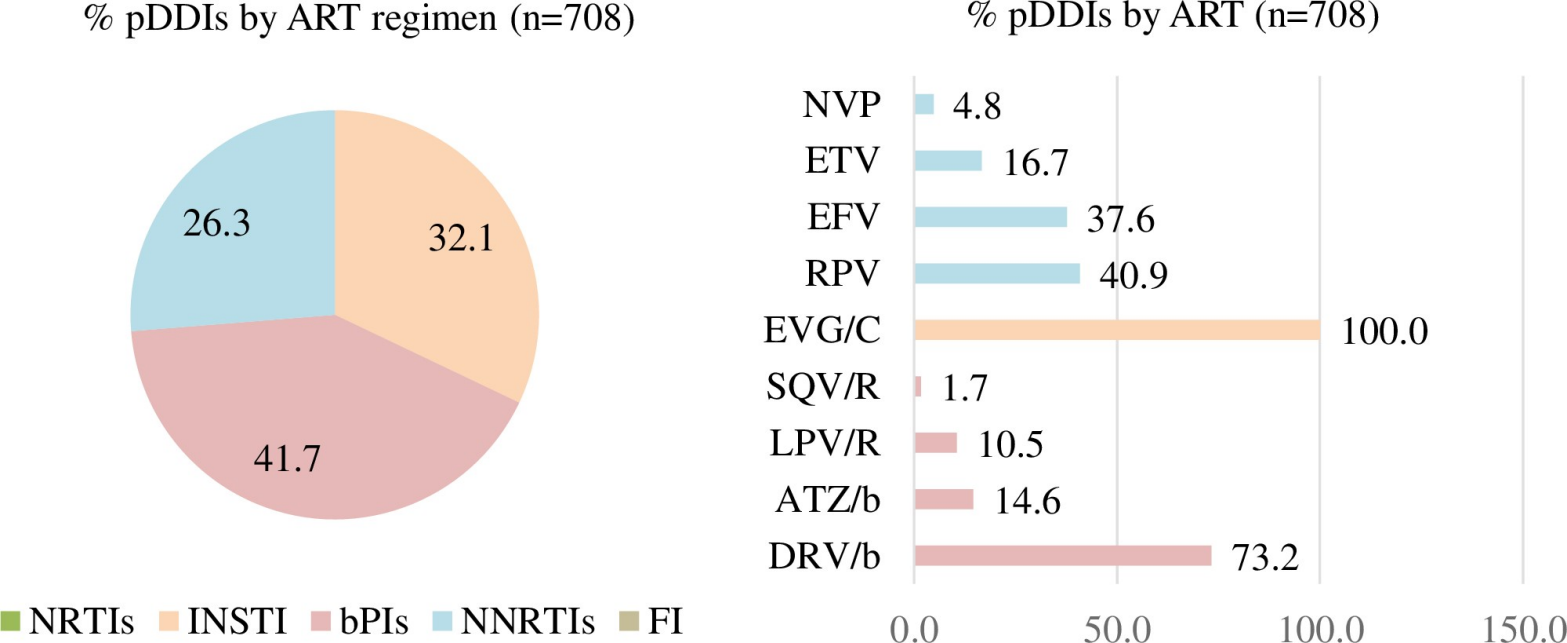
Prevalence of interactions per antiretroviral regimen. NRTIs: Nucleos(t)ide reverse transcriptase inhibitors; INSTI: Integrase strand transfer inhibitors; bPIs: Boosted-protease inhibitors; NNRTIs: Non-nucleos(t)ide reverse transcriptase inhibitors; FI: Fusion inhibitors; RPV: Rilpivirine; NVP: Nevirapine; ETV: Etravirine; EFV: Efavirenz; EVG/C: Elvitegravir/cobicistat; SQV/R: Saquinavir/ritonavir; LPV/R: Lopinavir/ritonavir; ATZ/b: Atazanavir boosted; DRV/b: Darunavir boosted; ART: Antiretroviral; pDDIs: Potential drug-drug interaction. n: number of pDDIs.

**Fig 4:**
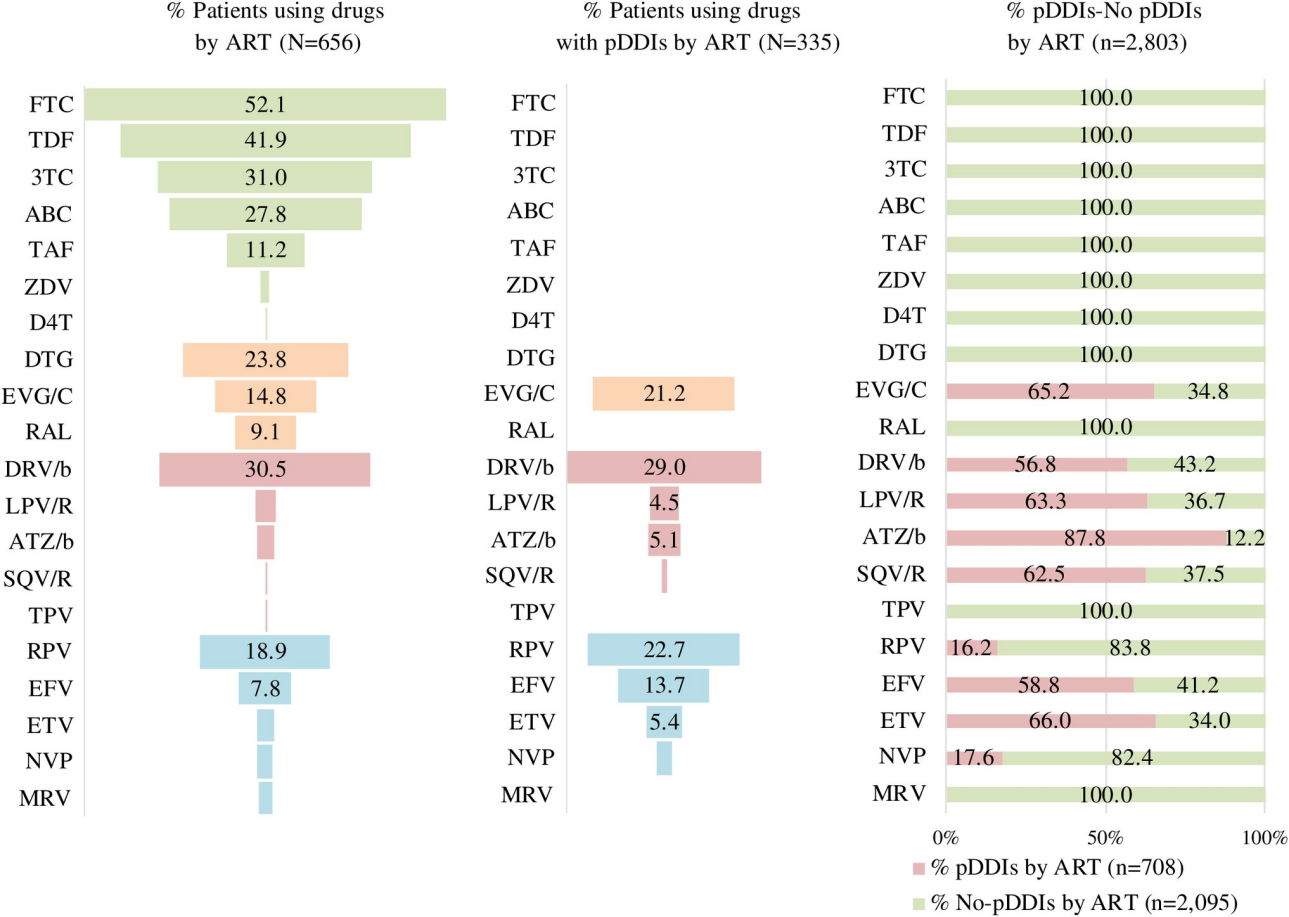
Prevalence of participants using drugs, with potential interactions and distribution per type of antiretroviral therapy. 3TC: Lamivudine; ABC: Abacavir; DTG: Dolutegravir; FTC: Emtricitabine; RPV: Rilpivirine; TDF: Tenofovir fumarate; TAF: Tenofovir alafenamide; EVG/C: Elvitegravir/cobicistat; EFV: Efavirenz; D4T: Stavudine; DRV/b: Darunavir boosted; RAL: Raltegravir; NVP: Nevirapine; TPV: Tipranavir; ETV: Etravirine; LPV/R: Lopinavir/ritonavir; MRV: Maraviroc; ATZ/b: Atazanavir boosted; ZDV: Zidovudine; SQV/R: Saquinavir/ritonavir; ART: Antiretroviral; pDDIs: Potential drug-drug interactions; No pDDIs: No potential drug-drug interaction; N: patients; n: number of pDDIs.

The presence of pDDIs differed according to the ART drug families used (Fig 3). Of the total number of pDDIs (n = 708), 41.7% resulted from regimens based on bPIs, 32.1% were caused by boosted INSTI, and 26.3% by NNRTIs. The bPIs associated with a higher prevalence of pDDIs was darunavir boosted with cobicistat or ritonavir. Within the INSTI family, elvitegravir boosted with cobicistat was the only drug leading to pDDIs. With regard to NNRTIs, the most frequent pDDIs were caused by rilpivirine and efavirenz. The only pDDI associated with a contraindicated combination was between cocaine and saquinavir. The higher pDDIs:non-pDDIs ratios in the ART category corresponded to boosted atazanavir (7.2:1), etravirine (1.9:1), and elvitegravir/cobicistat (1.9:1) (Fig 4).

The ART-illicit drug combinations that led to higher incidences of pDDIs were: boosted darunavir-cocaine, rilpivirine-cocaine, and elvitegravir/cobicistat-cocaine (Table 2).

**Table 2:** Potential interactions between illicit drugs and antiretroviral therapy.

	bPIs				NNRTIs				IIs
	DRV/b	ATZ/b	LPV/R	SQV/R	RPV	EFV	ETV	NVP	EVG/C
Cannabis	-	13	-	-	-	38	16	-	-
Cocaine	87	9	13	1	76	16	10	8	55
MDMA	27	-	3	1	-	-	-	-	39
GHB/GBL	24	3	2	1	-	-	-	-	33
Speed	20	5	5	1	-	-	-	-	25
Mephedrone	16	-	1	-	-	-	-	-	23
Ketamine	8	1	1	-	-	4	1	1	20
Methamphetamines	9	-	-	-	-	-	-	-	13
Heroin	17	7	6	-	-	5	3	-	7
LSD	5	2	-	1	-	2	1	-	9
Opium	3	-	-	-	-	-	-	-	3

Note: Results in the table refer to the number of participants with each interaction. No interactions were found in consumers of poppers. bPIs: Boosted-protease inhibitors; NNRTIs: Non-nucleos(t)ide reverse transcriptase inhibitors; RPV: Rilpivirine; NVP: Nevirapine; ETV: Etravirine; EFV: Efavirenz; EVG/C: Elvitegravir/cobicistat; SQV/R: Saquinavir/ritonavir; LPV/R: Lopinavir/ritonavir; ATZ/b: Atazanavir boosted. DRV/b: Darunavir boosted; MDMA: 3,4-Methylenedioxymethamfetamine; GHB/GBL: gamma-hydroxybutyrate/gamma-butyrolactone; LSD: Lysergic acid diethylamide.

### Prevalence-related differences and type of pDDIs according to participants’ drug consumption patterns

**Fig 5:**
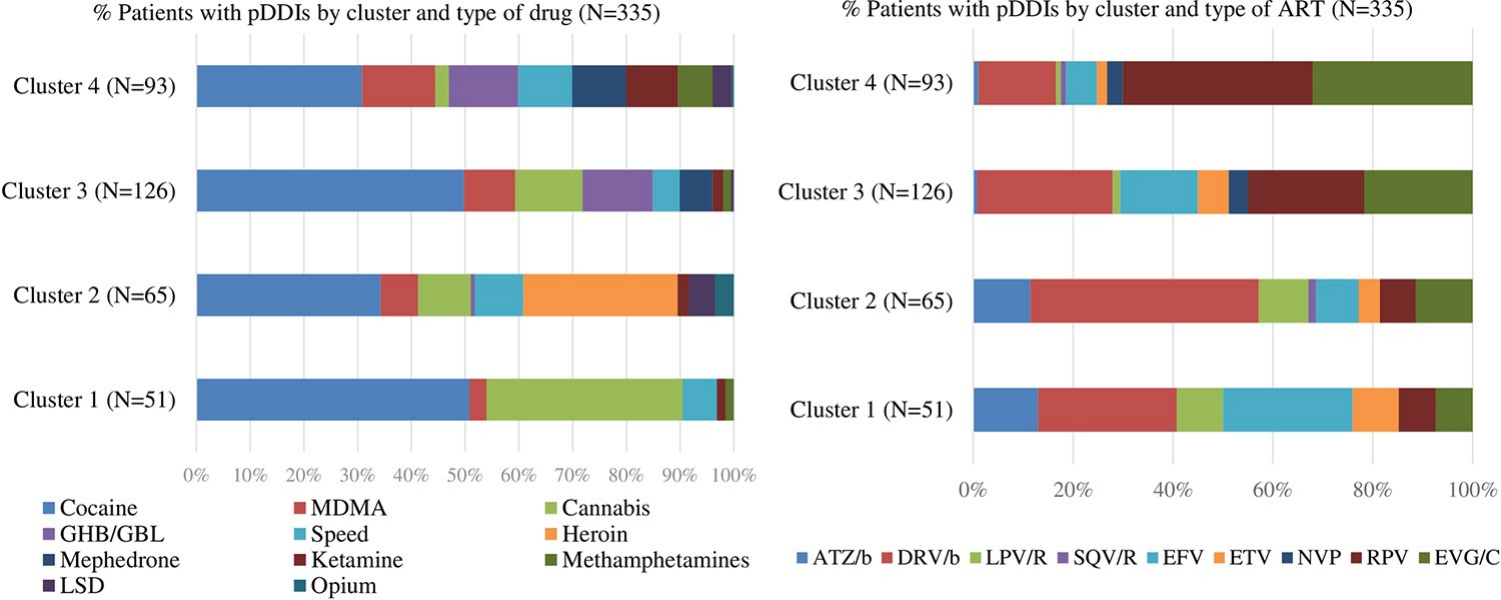
Prevalence of potential interactions per cluster and type of drug and antiretroviral therapy. MDMA: 3,4-Methylenedioxymethamfetamine; GHB/GBL: gamma-Hydroxybutyrate/gamma-Butyrolactone; LSD: Lysergic acid diethylamide; DRV/b: Darunavir boosted; RPV: Rilpivirine; EVG/C: Elvitegravir/cobicistat; EFV: Efavirenz; ETV: Etravirine; ATZ/b: Atazanavir boosted; LPV/R: Lopinavir/ritonavir; NVP: Nevirapine; SQV/R: Saquinavir/ritonavir; ART: Antiretroviral; pDDIs: Potential drug-drug interactions; N: Patients.

Prevalence and type of pDDIs were analysed according to the four illicit drug consumption patterns (clusters) found by Fuster-RuizdeApodaca et al. [20]. The consumption clusters identified were as follows: HTX consuming mainly cannabis [cluster 1 (C1), n = 160], HTX consuming mainly heroin and cocaine [cluster 2 (C2), n = 82], MSM exhibiting moderate illicit drug consumption [cluster 3 (C3), n = 265], and MSM with high rates of polydrug use [cluster 4 (C4), n = 149]. Statistically significant differences were found regarding pDDIs prevalence between patients in the different clusters: C2 (79.3%), C4 (62.4%), C3 (47.5%) and C1 (31.9%) (χ^2^ = 58.67, *p*<0.0001). The main types of illicit drugs involved in pDDIs were: cocaine and cannabis for C1; cocaine and heroin for C2; and cocaine for C3. C4 displayed a high rate of pDDIs for the majority of illicit drugs (Fig 5). pDDIs in the HTX clusters (C1 and C2) were mostly associated with bPI regimens (50.0% and 68.6%, respectively) whereas in the MSM clusters they were mostly related to NNRTIs (48.8% and 49.5%, respectively).

### Awareness, beliefs, intentional non-adherence, and communication with HIV specialists on the use of illicit drugs

As shown in Table 3, awareness among our sample of PLHIV who use illicit drugs regarding the potential of developing pDDIs when consuming illicit drugs together with their ART medication was slightly above the overall mean value. Also, the scores corresponding to the participants beliefs about the toxicity of pDDIs and to communication with healthcare providers about drug use were above the mean. However, the mean score for intentional non-adherence was lower than the mean value.

**Table 3:** Awareness and beliefs about interactions, intentional non-adherence, and communication with healthcare providers.

Dimensions (reliability)	Items in the questionnaire^a^	M±SD	PWpDDIs (n = 335)	PWOpDDIs (n = 321)	Student´s t^b^ (p value)
Awareness of drug-to-drug interactions (Cronbach’s α = 0.93, r = 0.88)	*• Are you aware that illicit drugs and HIV drugs may interact with one another*?*(1)*	3.09±1.21	3.07±1.22	3.11±1.21	-0.39 (0.693)
	*• Are you aware that taking your HIV medication together with illicit drugs may affect the outcome of your ART treatment*?*(2)*	3.12±1.21	3.10±1.23	3.15±1.19	-0.56 (0.570)
	**Awareness of potential pDDIs (composite score)**	**3.10±1.17**	**3.08±1.19**	**3.13±1.16**	-0.49 (0.619)
Beliefs about the toxicity of interactions (Cronbach’s α = 0.76, r = 0.61)	*• “I think that illicit drugs and HIV drugs should not be combined because the combination may be toxic” (3)*	2.95±1.41	2.93±1.39	2.97±1.44	-0.31 (0.755)
	*• “I think that taking illicit drugs at the same time as HIV medication may negatively impact my health”(4)*	2.86±1.40	2.92±1.39	2.79±1.40	1.19 (0.231)
	**Beliefs about pDDIs-induced toxicity (composite score)**	**2.90±1.26**	**2.92±1.24**	**2.88±1.28**	0.48 (0.625)
Intentional non-adherence behaviour (Cronbach’s α = 0.76)	*• “When I have taken illicit drugs*, *I wait for their effect to wear off before I take my HIV medication” (5)*	2.29±1.49	2.39±1.50	2.18±1.47	1.80 (0.071)
	*• “I try make sure I take my HIV medication and any illicit drugs at different times” (6)*	2.81±1.59	2.91±1.56	2.71±1.63	1.62 (0.104)
	*• “I’d rather not take my HIV medication when I know I am going to take illicit drugs” (7)*	1.50±1.04	1.64±1.17	1.34±0.86	3.77 (< .0001)
	*• “I usually refrain from taking my HIV medication when I’m under the effect of illicit drugs” (8)*	1.67±1.22	1.83±1.33	1.51±1.07	3.39 (0.001)
	**Intentional non-adherence behaviour (composite score)**	**2.06±1.00**	**2.19±1.04**	**1.93±0.94**	3.33 (0.001)
Communication with healthcare providers (Cronbach’s α = 0.83)	*• “I speak openly to my HIV doctor about my use of drugs without fear of being judged or criticized” (9)*	3.28±1.46	3.25±1.47	3.32±1.45	-0.58 (0.559)
	*• “I have told my HIV doctor what illicit drugs I use” (10)*	3.26±1.50	3.19±1.51	3.33±1.51	-1.23 (0.218)
	*• “My HIV doctor (or some other healthcare provider) has informed me about the potential effects of using drugs and taking my HIV medication at the same time” (11)*	3.01±1.48	2.92±1.50	3.10±1.45	-1.53 (0.126)
	*• “My HIV doctor (or some other healthcare provider) has advised me against mixing my HIV medication with illicit drugs because of pDDIs*.*” (12)*	2.82±1.54	2.73±1.53	2.92±1.55	-1.58 (0.114)
	**Communication with healthcare providers (composite score)**	**3.09±1.22**	**3.02±1.22**	**3.16±1.21**	-1.51 (0.129)

^a^Answers to the items were scored on a scale between 1 (’*not at all*’); 2 (’*little*’); 3 (’*something*’); 4 (’*quite a lot*’*)* and 5 (’*a lot*’).

^b^Measure of the importance de continuous variables (Student’s t test). PWpDDIs: patients with potential drug-drug interactions; PWOpDDIs: patients without potential drug-drug interactions. Variables in bold are the composite score of the items of each dimension.

A correlation analysis showed that an awareness of pDDIs was positively correlated with the belief that such pDDIs were toxic (*r* = 0.39, *p*<0.01) and with open communication with the healthcare provider about the use of drugs and potential pDDIs (*r* = 0.33, *p*<0.01). Beliefs about the toxicity of pDDIs exhibited a moderate-to-high association with intentional non-adherence to ART aimed at avoiding such pDDIs (*r* = 0.46, *p*<0.01).

### Influence of the awareness of pDDIs and the beliefs about their toxicity on intentional non-adherence in participants with pDDIs

The results of the ANCOVA analysis showed that PLHIV with pDDIs exhibited a significantly higher mean adjusted score than participants without pDDIs with respect to intentional non-adherence once the differences related to the awareness of pDDIs and beliefs about their toxicity covariates were controlled for (M = 2.18, SE = 0.048 vs. M = 1.94, SE = 0.048 respectively). Both covariates, pDDIs awareness and interactive toxicity beliefs, were significantly related to non-intentional adherence (Table 4).

**Table 4:** Analysis of covariance for non-intentional adherence in people living with HIV with interactions with knowledge and interactive toxicity beliefs as covariates.

Source	SS	df	MS	F	*p*	ɳ^2^
pDDIs Awareness (covariate)	13.49	1	13.49	17.62	<0.0001	0.03
Interactive toxicity beliefs (covariate)	152.29	1	152.29	198.89	<0.0001	0.23
pDDIs (group)	8.97	1	8.97	11.71	0.001	0.02
Error	499.26	652	0.77			
R^2^	0.249 (adjusted R^2^ = 0.245)					

SS = sum of squares; df = degrees of freedom; MS = Mean square; ɳ^2^ = partial eta squared.

### Differences between participants with and without pDDIs in health-related variables

No significant differences were found in the analysed self-reported health variables between the groups (Table 5).

**Table 5:** Differences in health-related variables between participants with or without potential interactions.

Variable^1^	PWpDDIs 51.1% (n = 335)	PWOpDDIs 48.9% (n = 321)	Statistical contrast
Immunological status (> 400 CD4/mm^*3*^*)*	86.7	83.0	χ^2^ = 1.43; *p* = 0.225
Undetectable viral load ^*a*^	93.8	94.5	χ^2^ = 0.16; *p* = 0.690
ART adherence^b^ (mean±SD)	84.65±11.22	86.09±9.38	t = -1.78*; p* = 0.078
WHOQoL score (mean±SD)	68.57±16.07	68.39±15.56	t *= 0*.*14*; *p* = 0.886
Visits to health centre (mean±SD)^c^	2.84±1.05	2.88±1.07	t *= -0*.*48; p* = 0.634
Visits to emergency room (mean±SD)^d^	1.73±0.87	1.77±0.83	t *= -0*.*04; p = 0*.*969*
Admissions (mean±SD)^d^	1.26±0.63	1.24±0.59	t *= -0*.*411; p = 0*.*681*

Note

^1^Data provided in percentages, except where specified.

χ^2^ Test (2xN tables) and t-test (continuous variables) to compare two stratified groups.

^a^Viral load category `*undetectable*´ was defined as <50 copies/ml.

^b^ART adherence by CEAT-VIH.

^c^Visits to health centre ranged from 1 (’*much less than usual*’); 2 (’*less than usual*’); 3 (’*same as usual*’); 4 (’*more than usual*’) and 5 (’*much more than usual*’*)*.

^d^Emergency visits and admissions ranged from 1 (’*never*’) to 5 (’*many times*’).

ART: antiretroviral therapy; PWpDDIs: patients with potential drug-drug interactions; PWOpDDIs: patients without potential drug-drug interactions.

## Discussion

This is the first study to evaluate the prevalence of pDDIs between ART therapy and illicit drugs, their distribution across patterns of illicit drug use, the implication of associated patients’ beliefs and their potential clinical impact in a national representative sample in Spain.

The study revealed that PLHIV using illicit drugs on ART, experienced a significant proportion of relevant pDDIs (51.1%). Garin et al. reported a similar prevalence (50%) of pDDIs with 208 PLHIV taking recreational drugs [27]. A study with 384 PLHIV who used recreational drugs in Southern Taiwan found potential pDDIs in 33.1% of participants [36]. Differences in prevalence are possibly due to differences in drug use patterns, differences in the ART regimens used in a specific area, and differences in the illicit drugs selected.

Our results showed that cocaine, cannabis and MDMA were the illicit drugs most frequently involved in pDDIs, with no interactions being observed among consumers of poppers. Regarding ART regimens, bPIs was the pharmacological family most frequently associated with pDDIs, followed by boosted INSTI and NNRTIs-based regimens. This is in line with the results obtained by Garin et al. [27] in Spain. In the ASTRA study, Daskalopoulou found similar results in 2,248 MSM with HIV in the UK [37]. Another study conducted in Taiwan found similar results [36], except for rilpivirine, which was characterised by a low pDDIs potential. In our study, rilpivirine accounted for 8.2% of pDDIs. According to the University of Liverpool Interaction Checker [18], the rilpivirine-cocaine combination could result in a pharmacokinetic interaction, which could lead to prolongation of the QT interval. Although the interaction database recommends caution, it warns that this DDI is very unlikely and that the quality of the evidence available is very low.

It should be mentioned that the likelihood of a DDI is different for each type of (therapeutic and/or illicit) drug. Consequently, drugs like cannabis, associated with high consumption rates, are associated with a very low (10.0%) pDDIs potential. On the other hand, other drugs such as opium or LSD are associated with a high risk of pDDIs (nearly 70%), although their use is much less prevalent.

As NRTIs are mainly excreted through the kidney and are not substrates of the CYP metabolic pathway, the prevalence of pDDIs associated with them is minimal [38]. Most relevant pDDIs between illicit drugs and ART regimens occur through the inhibition or induction of the CYP metabolic pathway (especially CYP3A4). A case in point is that of bPIs [14], where pDDIs often result in changes in (ART or illicit) drug concentrations [38, 39]. In our study, 97.3% of the pharmacokinetic pDDIs analysed would result in an accumulation of recreational drugs or their toxic metabolites, worsening the adverse effects of these drugs through the CYP metabolic pathway. The remaining 2.7% of pDDIs would tend to decrease plasma levels of ART drugs below the therapeutic range. Regarding pharmacodynamic pDDIs, 12.1% of the global pDDIs (atazanavir and rilpivirine with cocaine) could prolong the QT interval [18].

The Spanish GESIDA/PNS 2020 consensus document [40] and the US Department of Health and Human Services HIV treatment guidelines [41] recommend initiating therapy with a combination of two (dolutegravir/lamivudine) or three active ART medications. These ART regimens should comprise two NRTIs plus an INSTI, NNRTI, or bPI. These combinations have demonstrated similar rates of effectiveness in randomised clinical trials [42–44] but they exhibit variations regarding dosing frequency, number of pills a day, adverse events, genetic barriers to resistance, adherence, and potential pDDIs. Taking into consideration the widespread availability of illicit drugs and the high prevalence of illicit drug consumption found in our study, the best candidate for a third ART drug in PLHIV who use illicit drugs would be a NNRTI or non-boosted INSTI. The use of bPIs or boosted INSTI (elvitegravir/cobicistat) should be avoided in these cases due to their high prevalence of pDDIs. The highest pDDIs in the bPIs class corresponded to darunavir, to boosted elvitegravir in the INSTI class, and to rilpivirine in the NNRTIs class. Etravirine, doravirine, cabotegravir and bictegravir would be optimal candidates due to their low pDDIs profile. These ART were not included in this study because they are recently marketed. pDDIs should be addressed by a multidisciplinary team using state-of-the art regimens combined with rigorous monitoring, appropriate dosage adjustments, alternative forgiving therapeutic options for periods of non-adherence, and provision of information to patients about pDDIs [37].

To gain a better understanding of which PLHIV who use illicit drugs are at the highest risk of experiencing pDDIs, we analysed the pDDIs across the four clusters in Fuster Ruiz de Apodaca et al. [20]. PLHIV in C2 (users of mainly traditional intravenous drugs) were those with the highest prevalence of pDDIs, even though they used fewer drugs than PLHIV in C4, who presented with the highest rate of polydrug use. This could be because PLHIV in C2 mainly consumed heroin, which is associated with an elevated risk of pDDIs, and the fact that they were mainly under a bPI-based ART regimen as they had been HIV-positive for more than 20 years. bPIs are practically the only ART drugs that combine a higher genetic barrier to resistance, requiring a greater number of mutations to render treatment ineffective, with high robustness, where the selection of resistance in the case of virological failure is exceptional. This makes them the ART drugs with the highest permissiveness to non-adherence. C4 deserves special attention because these PLHIV could present with behaviours associated with chemsex, which tends to entail the consumption of higher doses and several substances at the same time, with a more intense effect and a higher risk of pDDIs.

The above-mentioned findings suggest that drug use and drug use patterns are among the most important considerations that clinicians must bear in mind when prescribing an ART regimen. Special attention should be paid to polydrug users because this is particularly prevalent in MSM with HIV who use drugs [20, 27, 45, 46]. Our analysis found many participants who engaged in polydrug use (66.5%), in line with other authors who also found high prevalence rates between 47.0 and 74.7% [27, 36, 45–47]. Chemsex, a term used to describe sexual relationships under the influence of certain psychoactive substances [48], has been related to polydrug use, high risk sexual practices, including condomless sex or slamming, and potential addiction [14, 48, 49]. This group should be given particular attention in order to minimise the risks derived from polydrug use, pDDIs, poor adherence, and high-risk sexual behaviours [27, 50].

Our study also investigated the participants awareness of potential pDDIs and their beliefs about toxicity that could result from them, as well as nature of the patient’s communication with healthcare providers about their use of illicit drugs. According to published studies, PLHIV with toxicity beliefs on interactions have a greater risk of deliberately missing doses when using drugs, which is associated with intentional non-adherence to ART [25]. Similarly, we found that the higher the awareness of the potential for pDDIs, the firmer the belief that toxicity could ensue. However, awareness was also associated with more open communication with healthcare providers. This is an aspect that was not evaluated in previous studies. Previous studies on that matter [25] did not explore whether respondents experienced pDDIs. Our study, after controlling for the effect of the awareness of potential pDDIs and the beliefs about the toxicity that they may cause, found that PLHIV with pDDIs presented with significantly more intentional non-adherence behaviours (anticipation, delays, or missing of ART when using drugs) than PLHIV without pDDIs. This points to the importance of promoting open clinician-patient communication. It is also necessary for healthcare providers to devote time to counselling, providing information, and establishing a trust-based doctor-patient relationship in order to promote an awareness of pDDIs and help patients to reject some toxicity-related beliefs that could lead to intentional non-adherence. To achieve this goal, it is paramount that healthcare providers should not maintain a judgmental attitude when addressing drug use.

It is important to note that unintentional adherence caused by low disease awareness, forgetfulness, temporary cognitive impairment or intoxication caused by drug consumption also contributes to lack of adherence [25]. Therefore, it is necessary to provide these people with adequate information on the effects of drugs and how to manage ART when using drugs to avoid forgetfulness and recognise the symptoms associated with the presence of intoxications. In this sense, social network-based interventions, with the creation of groups and communities and adequate social and health system support, can be very useful in understanding and predicting behaviours related to drug intoxication.

Many PLHIV, especially the older subpopulation group, suffer from co-morbidities, including psychiatric disorders and cognitive decline [51]. Comorbidities are, in turn, associated with a higher degree of polypharmacy, including drugs such as antidepressants, antipsychotics and other medicines with anticholinergic properties that increase the risk of cognitive impairment [52]. Adherence and, subsequently, the efficacy of treatment may be compromised. This may be especially relevant in the people using illicit drugs due to the synergic impact on cognitive functioning. The need of appropriate assessment of anticholinergic burden and cognitive impact due to polypharmacy and comorbidities should be considered in clinical practice.

Although PLHIV with pDDIs usually present with a higher prevalence of intentional non-adherence, their clinical health variables were not affected in our sample. We did not find any significant differences between PLHIV with and without pDDIs in terms of viral load, CD4 count, quality of life or number of visits to a health centre. As our data are cross-sectional, cohort studies are necessary to investigate whether pDDIs could have a long-term impact on the health of PLHIV.

Our study has several limitations. Firstly, its cross-sectional nature does not allow causal relationships to be established. Secondly, the data presented come from a self-reported questionnaire, which precludes verification of the veracity of the information. Respondents may well have attenuated their responses regarding drug use, leading to a bias of information. Indeed, underreporting is likely to occur in studies of this kind, especially when it comes to drugs that enjoy less social acceptance such as the newest or strongest drugs. Thirdly, our results correspond to our geographical area in Spain. Comparisons across different studies are problematic because of the great variability in terms of inclusion criteria, different recall periods (e.g. last month, last year), recreational drugs selected, drug patterns, age range, and sexual orientation. Fourthly, another possible limitation of this study is that we established our pDDIs on the basis of a database that provided expected pDDIs and did not monitor the adverse clinical outcomes potentially arising from these pDDIs. Finally, ART prescribing patterns change with time as new drugs and evidence is available. The increase in INSTI-based regimens and the use of dual therapies may have some impact in terms of pDDIs prevalence. However, the relevance of eliminating NRTIs may have a very limited impact as this pharmacological group does not have relevant theoretical interactions with illicit drugs.

## Conclusions

The present study addresses a critical gap in the understanding of the prevalence of pDDIs among PLHIV who use illicit drugs in Spain and their impact on health outcomes. Our findings suggest that the prevalence of potentially relevant pDDIs is significant in PLHIV who use drugs. It also shows that the prevalence of pDDIs differs according to the drug use pattern considered, with polydrug users being those at the highest risk. Moreover, our results show that experiencing pDDIs could lead to intentional non-adherence. Although we did not find any impact of pDDIs on other health-related variables, longitudinal studies should be undertaken to determine whether pDDIs could have an impact on the long-term health status of PLHIV.

Our study provides indicators for selecting PLHIV at a higher risk of pDDIs according to their drug use pattern. Such PLHIV could greatly benefit from having their treatment monitored. An awareness, recognition, and the correct management of pDDIs are important in optimising the medical and pharmaceutical care administered to PLHIV and could help to prevent a loss of efficacy of the drugs administered as well as any adverse events that they may cause. Therefore, it is essential to provide practitioners responsible for the care of patients with adequate training and the support tools required to easily access validated information.

## Abbreviations

ART: antiretroviral therapy
PLHIV: people living with human immunodeficiency virus
pDDIs: drug-drug interactions
bPIs: boosted-protease inhibitor-based regimens
NNRTIs: non-nucleoside/nucleotide reverse transcriptase inhibitors
IIs: integrase inhibitor
INSTI: integrase strand transfer inhibitors
NRTIs: nucleoside/nucleotide reverse transcriptase inhibitors
MSM: men who have sex with men
HTX: heterosexual
STIs: sexually transmitted infections

## References

[1] ONUSIDA. Hoja informativa: últimas estadísticas sobre el estado de la epidemia de sida (datos a cierre de 2019). 2020. Availablefrom: https://www.unaids.org/sites/default/files/media_asset/UNAIDS_FactSheet_es.pdf

[2] Unidad de vigilancia del VIH, ITS y hepatitis. Actualización del Continuo de Atención del VIH en España, 2017–2019. Madrid: Centro Nacional de Epidemiología–Instituto de Salud Carlos III / Plan Nacional sobre el Sida–Dirección General de Salud Pública; 2020. Available from: https://www.mscbs.gob.es/ciudadanos/enfLesiones/enfTransmisibles/sida/vigilancia/ESTIMACION_DEL_CONTINUO_DE_ATENCIoN_DEL_VIH_EN_ESPAnA_Nov2020.pdf

[3] Jakeman B, Nasiri M, Ruth L, Morse C, Mahatme S, Patel N. Comparing the frequencies of contraindicated drug-drug interactions between differing antiretroviral regimens in HIV-infected patients. Ann Pharmacother. 2017; 51(5): 365–72. doi: 10.1177/1060028016685115 28367698

[4] Oreagba IA, Usman SO, Oshikoya KA, Akinyede A, Agbaje E, Opanuga O, et al. Clinically significant drug-drug interaction in a large antiretroviral treatment centre in Lagos, Nigeria. J Popul Ther Clin Pharmacol. 2019 Jan 22; 26(1): e1–e19. doi: 10.22374/1710-6222.26.1.1 31002484

[5] Rajasingham R, Mimiaga MJ, White JM, Pinkston MM, Baden RP, Mitty JA. A systematic review of behavioural and treatment outcome studies among HIV-infected men who have sex with men who abuse crystal methamphetamine. AIDS Patient Care STDS. 2012 Jan; 26(1): 36–52. doi: 10.1089/apc.2011.0153 22070609 PMC3248609

[6] Degenhardt L, Mathers B, Guarinieri M, Panda S, Phillips B, Strathdee SA, et al. Meth/amphetamine use and associated HIV: Implications for global policy and public health. Int J Drug Policy. 2010 Sep; 21(5): 347–58. doi: 10.1016/j.drugpo.2009.11.007 20117923

[7] Cascorbi I. Drug interactions—principles, examples and clinical consequences. Dtsch Arztebl Int. 2012 Aug; 109(33–34): 546–55; quiz 556. doi: 10.3238/arztebl.2012.0546 23152742 PMC3444856

[8] Baecke C, Gyssens IC, Decoutere L, van der Hilst JCH, Messiaen P. Prevalence of drug-drug interactions in the era of HIV integrase inhibitors: a retrospective clinical study. Neth J Med. 2017 Jul; 75(6): 235–240. 28741582

[9] Shapiro LE, Shear NH. Drug interactions: Proteins, pumps, and P-450s. J Am Acad Dermatol. 2002 Oct;47(4): 467–84; quiz 485–8. doi: 10.1067/mjd.2002.126823 12271287

[10] Priyanka PSSL, Varma DM, Immadisetti K, Rajesh R, Vidyasagar S, Guddattu V. Recognition of possible risk factors for clinically significant drug-drug interactions among Indian people living with HIV receiving highly active antiretroviral therapy and concomitant medications. Int J Risk Safety Med. 2017; 29(1–2): 25–55. doi: 10.3233/JRS-170738 28885219

[11] Yiu P, Nguyen NN, Holodniy M. Clinically significant drug interactions in younger and older human immunodeficiency virus-positive patients receiving antiretroviral therapy. Pharmacotherapy. 2011 May; 31(5): 480–9. doi: 10.1592/phco.31.5.480 21923429

[12] Iniesta-Navalón C, Franco-Miguel JJ, Gascón-Cánovas JJ, Rentero-Redondo L. Identification of potential clinically significant drug interactions in HIV-infected patients: a comprehensive therapeutic approach. HIV Med. 2015 May; 16(5): 273–9. doi: 10.1111/hiv.12205 25523089

[13] Patel N, Abdelsayed S, Veve M, Miller CD. Predictors of clinically significant drug-drug interactions among patients treated with non-nucleoside reverse transcriptase inhibitor-, protease inhibitor-, and raltegravir-based antiretroviral regimens. Ann Pharmacother. 2011 Mar; 45(3): 317–24. doi: 10.1345/aph.1P576 21386025

[14] Bracchi M, Stuart D, Castles R, Khoo S, Back D, Boffito M. Increasing use of ’party drugs’ in people living with HIV on antiretrovirals: a concern for patient safety. AIDS. 2015 Aug 24; 29(13): 1585–92. doi: 10.1097/QAD.0000000000000786 26372268

[15] Kumar S, Rao PS, Earla R, Kumar A. Drug-drug interactions between anti-retroviral therapies and drugs of abuse in HIV systems. Expert Opin Drug Metab Toxicol. 2015 Mar; 11(3): 343–55. doi: 10.1517/17425255.2015.996546 25539046 PMC4428551

[16] Kumar S, Rao PS, Earla R, Kumar A. Drug-drug interactions between anti-retroviral therapies and drugs of abuse in HIV systems. Expert Opin Drug Metab Toxicol. 2015 Mar; 11(3): 343–55. doi: 10.1517/17425255.2015.996546 25539046 PMC4428551

[17] Antoniou T, Tseng AL. Interactions between recreational drugs and antiretroviral agents. Ann Pharmacother. 2002 Oct; 36(10): 1598–613. doi: 10.1345/aph.1A447 12243611

[18] Liverpool HIV Pharmacology Group (LHPG). HIV drug interactions webpage. http://www.hiv-druginteractions.org/.

[19] Serrano López de las Hazas J.I. Interacciones farmacológicas de los nuevos antirretrovirales. Farm Hosp. 2011; 35(1): 36–43. ISSN 1130-6343. doi: 10.1016/j.farma.2010.01.018 21208819

[20] Fuster-RuizdeApodaca MJ, Castro-Granell V, Garin N, Laguía A, Jaén Á, Iniesta C, et al. Prevalence and patterns of illicit drug use in people living with HIV in Spain: A cross-sectional study. PLoS One. 2019 Jun 17; 14(6): e0211252. doi: 10.1371/journal.pone.0211252 31206550 PMC6576760

[21] Pirmohamed M. Drug-drug interactions and adverse drug reactions: separating the wheat from the chaff. Wien Klin Wochenschr. 2010 Feb; 122(3–4): 62–4. doi: 10.1007/s00508-010-1309-1 20213370

[22] Zhou J, Shaw SG, Gilleece Y. Dilated common bile duct and deranged liver function tests associated with ketamine use in two HIV-positive MSM. Int J STD AIDS. 2013 Aug; 24(8): 667–9. doi: 10.1177/0956462413479894 23970577

[23] Henry JA, Hill IR. Fatal interaction between ritonavir and MDMA. Lancet. 1998 Nov 28; 352(9142): 1751–2. doi: 10.1016/s0140-6736(05)79824-x 9848354

[24] Hales G, Roth N, Smith D. Possible fatal interaction between protease inhibitors and methamphetamine. Antivir Ther. 2000 Mar; 5(1): 19. 10846588

[25] Kalichman SC, Kalichman MO, Cherry C, Hoyt G, Washington C, Grebler T, et al. Intentional medication non-adherence because of interactive toxicity beliefs among HIV-positive active drug users. J Acquir Immune Defic Syndr. 2015; 70(5): 503–9. doi: 10.1097/QAI.0000000000000776 26226250 PMC4648658

[26] Thompson MA, Aberg JA, Hoy JF, Telenti A, Benson C, Cahn P, et al. Antiretroviral treatment of adult HIV infection: 2012 recommendations of the International Antiviral Society-USA panel. JAMA. 2012 Jul 25; 308(4): 387–402. doi: 10.1001/jama.2012.7961 22820792

[27] Garin N, Zurita B, Velasco C, Feliu A, Gutierrez M, Masip M, et al. Prevalence and clinical impact of recreational drug consumption in people living with HIV on treatment: a cross-sectional study. BMJ Open. 2017 Jan 18; 7(1): e014105. doi: 10.1136/bmjopen-2016-014105 28100565 PMC5253545

[28] Fuster-RuizdeApodaca MJ, Castro-Granell V, Laguía A, Jaén Á, Cenoz S, Galindo MJ. Drug use and antiretroviral therapy (ART) interactions: a qualitative study to explore the knowledge, beliefs, adherence, and quality of life of people living with HIV taking ART and illicit drugs. AIDS Res Ther. 2020 May 24; 17(1): 24. doi: 10.1186/s12981-020-00279-y 32448214 PMC7245822

[29] HIV/AIDS Treatment Adherence, Health Outcomes and Cost Study Group. The HIV/AIDS Treatment Adherence, Health Outcomes and Cost Study: conceptual foundations and overview. AIDS Care. 2004; 16 Suppl 1: S6–21. doi: 10.1080/09540120412331315312 15739266

[30] Cashman SB, Adeky S, Allen AJ 3rd, Corburn J, Israel BA, Montaño J, et al. The power and the promise: working with communities to analyse data, interpret findings, and get to outcomes. Am J Public Health. 2008 Aug; 98(8): 1407–17. doi: 10.2105/AJPH.2007.113571 18556617 PMC2446454

[31] Wallerstein NB, Duran B. Using community-based participatory research to address health disparities. Health Promot Pract. 2006 Jul; 7(3): 312–23. doi: 10.1177/1524839906289376 16760238

[32] Kalichman SC, Grebler T, Amaral CM, McNerey M, White D, Kalichman MO, et al. Intentional non-adherence to medications among HIV positive alcohol drinkers: prospective study of interactive toxicity beliefs. J Gen Intern Med. 2013 Mar; 28(3): 399–405. doi: 10.1007/s11606-012-2231-1 23065532 PMC3579979

[33] Remor E. Avaliação on-line da adesão ao tratamento antirretroviral para a infecção pelo HIV. Paper session presented at 11o. Congresso Nacional De Psicologia da Saúde. Lisboa, Portugal; 2016.

[34] Fuster-RuizdeApodaca MJ, Laguía A, Safreed-Harmon K, Lazarus JV, Cenoz S, Del Amo J. Assessing quality of life in people with HIV in Spain: psychometric testing of the Spanish version of WHOQOL-HIV-BREF. Health Qual Life Outcomes. 2019 Aug 19; 17(1): 144. doi: 10.1186/s12955-019-1208-8 31426799 PMC6700970

[35] Pedroso B, Pilatti LA, de Francisco AC, dos Santos CB. Quality of life assessment in people with HIV: analysis of the WHOQOL-HIV syntax. AIDS Care. 2010 Mar; 22(3): 361–72. doi: 10.1080/09540120903111502 20390517

[36] Chen GL, Lin SY, Lo HY, Wu HC, Lin YM, Chen TC, et al. Clinical impact of recreational drug use among people living with HIV in southern Taiwan. J Microbiol Immunol Infect. 2020 Aug 12: S1684-1182(20): 30172–9. doi: 10.1016/j.jmii.2020.07.016 32847749

[37] Daskalopoulou M, Rodger AJ, Phillips AN, Speakman A, Lampe FC. Prevalence of recreational drug use is indiscriminate across antiretroviral regimens of differing drug-drug interactions among MSM. AIDS. 2016 Mar 13; 30(5): 810–2. doi: 10.1097/QAD.0000000000000994 26913713

[38] Staltari O, Leporini C, Caroleo B, Russo E, Siniscalchi A, De Sarro G, et al. Drug-drug interactions: antiretroviral drugs and recreational drugs. Recent Pat CNS Drug Discov. 2014; 9(3): 153–63. doi: 10.2174/1574889809666141127101623 25429704

[39] Abbott KL, Flannery PC, Gill KS, Boothe DM, Dhanasekaran M, Mani S, et al. Adverse pharmacokinetic interactions between illicit substances and clinical drugs. Drug Metab Rev. 2020 Feb; 52(1): 44–65. doi: 10.1080/03602532.2019.1697283 31826670

[40] Documento de consenso de Gesida/plan Nacional sobre el Sida respecto al tratamiento antirretroviral en adultos infectados por el virus de la inmunodeficiencia humana (Actualización enero 2019). Panel de Expertos de GeSIDA y Plan nacional sobre el Sida. Ministerio de Sanidad, Consumo y Bienestar Social.

[41] Panel on Antiretroviral Guidelines for Adults and Adolescents. Guidelines for the Use of Antiretroviral Agents in Adults and Adolescents with HIV. Department of Health and Human Services. Available at http://www.aidsinfo.nih.gov/ContentFiles/AdultandAdolescentGL.pdf. Accessed [4 April 2021].

[42] Haubrich RH, Riddler SA, DiRienzo AG, Komarow L, Powderly WG, Klingman K, et al. Metabolic outcomes in a randomized trial of nucleoside, non-nucleoside and protease inhibitor-sparing regimens for initial HIV treatment. AIDS. 2009 Jun 1; 23(9): 1109–18. doi: 10.1097/QAD.0b013e32832b4377 19417580 PMC2739977

[43] Lennox JL, Dejesus E, Berger DS, Lazzarin A, Pollard RB, Ramalho Madruga JV, et al. Raltegravir versus Efavirenz regimens in treatment-naive HIV-1-infected patients: 96-week efficacy, durability, subgroup, safety, and metabolic analyses. JAcquir Immune Defic Syndr. 2010 Sep; 55(1): 39–48. Erratum in: J Acquir Immune Defic Syndr. 2011 Dec 1; 58(4): e120. Dosage error in article text. doi: 10.1097/QAI.0b013e3181da1287 20404738 PMC6065510

[44] Puls RL, Srasuebkul P, Petoumenos K, Boesecke C, Duncombe C, Belloso WH, et al. Efavirenz versus boosted atazanavir or zidovudine and abacavir in antiretroviral treatment-naive, HIV-infected subjects: week 48 data from the Altair study. Clin Infect Dis. 2010 Oct 1; 51(7): 855–64. doi: 10.1086/656363 20735258

[45] Daskalopoulou M, Rodger A, Phillips AN, Sherr L, Speakman A, Collins S, et al. Recreational drug use, polydrug use, and sexual behaviour in HIV-diagnosed men who have sex with men in the UK: results from the cross-sectional ASTRA study. Lancet HIV. 2014 Oct; 1(1): e22–31. doi: 10.1016/S2352-3018(14)70001-3 26423813

[46] Schmidt AJ, Bourne A, Weatherburn P, Reid D, Marcus U, Hickson F, et al. Illicit drug use among gay and bisexual men in 44 cities: Findings from the European MSM Internet Survey (EMIS). Int J Drug Policy. 2016 Dec;38:4–12. doi: 10.1016/j.drugpo.2016.09.007 27788450

[47] Mor Z, Turner D, Livnat Y, Levy, I. Recreational drug and excessive alcohol use among HIV-infected men who have sex with men in Central Israel. BMC Public Health 2019; 19: 1360. doi: 10.1186/s12889-019-7747-4 31651293 PMC6813972

[48] Bourne A, Reid D, Hickson F, Torres-Rueda S, Weatherburn P. Illicit drug use in sexual settings (’chemsex’) and HIV/STI transmission risk behaviour among gay men in South London: findings from a qualitative study. Sex Transm Infect. 2015 Dec;91(8):564–8. doi: 10.1136/sextrans-2015-052052 26163510

[49] Garin N, Velasco C, De Pourcq JT, Lopez B, Gutierrez Mdel M, Haro JM, et al. Recreational drug use among individuals living with HIV in Europe: review of the prevalence, comparison with the general population and HIV guidelines recommendations. Front Microbiol. 2015 Jul 14;6:690. doi: 10.3389/fmicb.2015.00690 26236288 PMC4500990

[50] Grabovac I, Meilinger M, Schalk H, Leichsenring B, Ernst T, et al. Prevalence and associations of illicit drug and polydrug use in people living with HIV in Vienna. Sci Rep 2018; 8: 8046. doi: 10.1038/s41598-018-26413-5 29795303 PMC5966416

[51] Back D, Marzolini C. The challenge of HIV treatment in an era of polypharmacy. J Int AIDS Soc. 2020 Feb;23(2):e25449. doi: 10.1002/jia2.25449 . 32011104 PMC6996317

[52] Livio F, Marzolini C. Prescribing issues in older adults living with HIV: thinking beyond drug-drug interactions with antiretroviral drugs. Ther Adv Drug Saf. 2019 Oct 3;10:2042098619880122. doi: 10.1177/2042098619880122 31620274 PMC6777047

